# Summary Report of the SNMMI AI Task Force Radiomics Challenge 2024

**DOI:** 10.2967/jnumed.124.269425

**Published:** 2025-08

**Authors:** Ronald Boellaard, Arman Rahmim, Jacoba J. Eertink, Ulrich Duehrsen, Lars Kurch, Pieternella J. Lugtenburg, Sanne E. Wiegers, Gerben J.C. Zwezerijnen, Josée M. Zijlstra, Martijn W. Heymans, Irène Buvat

**Affiliations:** 1 Department of Radiology and Nuclear Medicine, Amsterdam UMC, Cancer Center Amsterdam, Amsterdam, The Netherlands;; 2 Departments of Radiology and Physics, University of British Columbia, Vancouver, British Columbia, Canada;; 3 Department of Hematology, West German Cancer Center, University Hospital Essen, University of Duisburg-Essen, Essen, Germany;; 4 Clinic and Polyclinic for Nuclear Medicine, Department of Nuclear Medicine, University of Leipzig, Leipzig, Germany;; 5 Department of Hematology, Erasmus MC Cancer Institute, University Medical Center Rotterdam, Rotterdam, The Netherlands;; 6 Department of Hematology, Amsterdam UMC, Cancer Center Amsterdam, Amsterdam, The Netherlands;; 7 Department of Epidemiology and Data Science, Amsterdam Public Health Research Institute, Amsterdam UMC, Amsterdam, The Netherlands; and; 8 Institut Curie, Université PSL, Laboratoire d’Imagerie Translationnelle en Oncologie, Orsay, France

**Keywords:** radiomics, machine learning, PET, prognosis, lymphoma

## Abstract

In medical imaging, challenges are competitions that aim to provide a fair comparison of different methodologic solutions to a common problem. Challenges typically focus on addressing real-world problems, such as segmentation, detection, and prediction tasks, using various types of medical images and associated data. Here, we describe the organization and results of such a challenge to compare machine-learning models for predicting survival in patients with diffuse large B-cell lymphoma using a baseline ^18^F-FDG PET/CT radiomics dataset. **Methods:** This challenge aimed to predict progression-free survival (PFS) in patients with diffuse large B-cell lymphoma, either as a binary outcome (shorter than 2 y versus longer than 2 y) or as a continuous outcome (survival in months). All participants were provided with a radiomic training dataset, including the ground truth survival for designing a predictive model and a radiomic test dataset without ground truth. Figures of merit (FOMs) used to assess model performance were the root-mean-square error for continuous outcomes and the C-index for 1-, 2-, and 3-y PFS binary outcomes. The challenge was endorsed and initiated by the Society of Nuclear Medicine and Molecular Imaging AI Task Force. **Results:** Nineteen models for predicting PFS as a continuous outcome from 15 teams were received. Among those models, external validation identified 6 models showing similar performance to that of a simple general linear reference model using SUV and total metabolic tumor volumes (TMTV) only. Twelve models for predicting binary outcomes were submitted by 9 teams. External validation showed that 1 model had higher, but nonsignificant, C-index values compared with values obtained by a simple logistic regression model using SUV and TMTV. **Conclusion:** Some of the radiomic-based machine-learning models developed by participants showed better FOMs than did simple linear or logistic regression models based on SUV and TMTV only, although the differences in observed FOMs were nonsignificant. This suggests that, for the challenge dataset, there was limited or no value seen from the addition of sophisticated radiomic features and use of machine learning when developing models for outcome prediction.

PET/CT using ^18^F-FDG plays a critical role in oncology and hematology as a diagnostic, prognostic, and predictive tool (1). Moreover, ^18^F-FDG PET/CT is used to assess response to treatment. Quantitative metrics, such as SUVs and total metabolic tumor volumes (TMTVs), are increasingly used in clinical research (2). For example, changes in SUVs are still used to assess response to treatment, though the first PET-based response criteria were introduced a decade ago (3).

More-extensive phenotyping of ^18^F-FDG uptake in and across lesions can be obtained by analyzing radiomic features (4,5). Radiomic features offer comprehensive quantitative data on tumor phenotype, including morphology, texture, dissemination patterns, and intensity, and may provide insight into the underlying biologic characteristics of tumors. To facilitate the use of radiomic features, standardization of radiomic feature calculations and processing has been proposed by the International Biomarker Standardization Initiative (6). In addition, statistical or machine-learning models using standardized radiomic features have shown value in predicting disease progression, treatment response, and outcomes (5,7–9).

One of the main challenges in developing radiomics-based models is the multitude of radiomic features and the relatively small datasets (<500) available. In addition, the distribution of the dataset (e.g., outcome data) can be imbalanced, which hampers model development, training, and validation. Moreover, many features tend to be highly correlated due to mathematic equivalence or underlying biologic associations. The overdimensionality of the feature space, the redundancy among features, and the small datasets pose several challenges for model development (10).

Model development typically requires several steps, such as dimensionality reduction, feature normalization to address the large variability in feature value ranges, selection of the most optimal model algorithm, and choice of a method to address data imbalance during training and evaluation (11,12). The latter may also include the selection of the best figure of merit (FOM) (i.e., loss function) to assess model performance during training or validation. For each of these steps, many methods are available, and many settings need to be optimized. Often, investigators perform an extensive grid search over methods and settings, thereby increasing the chance of optimal model performance. External validation using data unseen during model development and training is of utmost importance to validate models, as recommended in Society of Nuclear Medicine and Molecular Imaging AI Task Force (SNMMI-AITF) guidelines (13,14).

The many methods, options, and settings available during model development make it difficult to determine the optimal model. Therefore, the SNMMI-AITF proposed a radiomics challenge using a radiomic feature dataset in machine-learning models. The aim of the challenge was 2-fold: obtain an understanding of the various approaches and methods used by machine-learning experts when developing machine-learning models and determine if sophisticated machine-learning models based on radiomic features could outperform models based on standard PET metrics, such as SUV and TMTV. To this end, a radiomic feature dataset (containing no images) with survival outcome was provided to each participant for training, and a second dataset without outcome data was provided for external testing. Here, we provide a summary of the challenge method and results and report on the variety of approaches used during the model development process.

## MATERIALS AND METHODS

### Setup and Timeline of the SNMMI-AITF Challenge

The SNMMI-AITF radiomics challenge was launched on July 1, 2023, as part of SNMMI-AITF activities. Before launching the challenge, a website (https://sites.snmmi.org/Therapy/SNMMI-THERAPY/AI_Challenge.aspx) was created to announce the challenge and provide background information on the challenge itself, datasets for training and testing, FOMs for evaluating model performance, timelines, and the terms and conditions for participation. Parties or individuals could express their interest in participation through the website, and a register of interested participants was created and tracked by SNMMI. Registered participants received the training dataset by the end of October 2023, including a data format description. Participants were allowed to train their model until January 2024, but the external testing dataset was provided in December 2023. In early 2024, participants were requested to provide their model predictions for the external dataset to challenge team members, who assessed model performances without knowledge of participants’ identities. Results regarding model performance were announced and winners of the challenge notified in March 2024. Challenge winners presented their results at the SNMMI Annual Meeting in June 2024.

### Description of the Datasets

The training and testing datasets were provided as an Excel (Microsoft) sheet including 504 ^18^F-FDG PET/CT radiomic features, including SUV_peak_ (defined as the maximum SUV_peak_ across all lesions) and TMTVs. A list of all features is provided in the supplemental materials, available at http://jnm.snmjournals.org. All other features were renamed to prevent the disclosure of possible useful features mentioned in prior publications (15–17) and to ensure that a fully data-driven approach would be followed during model development.

The training dataset, previously used by Ferrández et al. (18), contained radiomic features from 296 patients. The training dataset was derived from the multicenter HOVON-84 trial in diffuse large B-cell lymphoma (19). The external testing dataset, including data from 340 patients, was used to assess model performance and was derived from the multicenter PETAL study (20). These datasets were chosen to allow comparison of challenge findings with those in Ferrández et al. (18), where deep-learning models as well as models based on PET uptake measures with and without the use of clinical information were used. In both trials, patients received several cycles of either standard rituximab, cyclophosphamide, doxorubicin, vincristine, and prednisone treatment or treatment with intensified rituximab, cyclophosphamide, doxorubicin, vincristine, and prednisone. There were no differences in survival between treatment groups in both studies; therefore, we included all patients within the training and testing datasets, as was done previously (21). The studies were approved by the institutional review board, and all participants gave written informed consent to participate and share the data. The datasets were fully anonymized.

The training radiomic dataset was derived from an earlier study (18). Along with the radiomic features, progression-free survival (PFS) (in months) and event type (event or no event [i.e., study drop out or end of follow-up]) were provided for each patient in the training dataset. The datasets did not contain any patient demographics or clinical information, as we previously found that excluding this information did not notably decrease outcome prediction performance (18,21). The testing dataset contained only radiomics features (no outcome data). A description of the format of the training dataset was provided to participants (supplemental materials).

For both the training and testing datasets, the radiomics features were calculated using an International Biomarker Standardization Initiative–compliant RaCaT tool (22) from ^18^F-FDG PET images and lesion volumes of interest (VOIs). Lesion VOIs were defined using the international benchmark threshold (SUV of 4) for all visible lesions in the PET images (23). All VOIs were reviewed by an experienced nuclear medicine physician. Images were resampled to 2 × 2 × 2 mm voxel size using centered-grid trilinear interpolation before radiomic feature extraction. To calculate textural features, all VOIs in a patient were considered as a single region (possibly including several unconnected components), and images were discretized with a fixed SUV bin size of 0.25 g/mL. Texture features were based on the gray-level cooccurrence matrix, gray-level run-length matrix, gray-level size-zone matrix, gray-level distance-zone matrix, neighborhood gray-tone difference matrix, and neighboring gray-level dependence matrix, with up to 8 matrix calculation methods. In addition, 4 dissemination features were extracted at the patient level to quantify the distance between lesions, as suggested by Cottereau et al. (7), and the number of lesions. Ten interlesion heterogeneity features were extracted to quantify the differences in intensity between lesions, and 3 features were extracted to quantify the differences in volume between lesions (15,17).

### Model Performance Assessment and FOMs

Participants could participate in 2 challenges. One challenge aimed at predicting continuous outcomes (continuous model), and the second challenge focused on predicting dichotomous outcomes (i.e., a classification model to predict PFS being shorter or longer than 1, 2, and 3 y). Participants were allowed to provide up to 2 results based on different models for both continuous and dichotomous outcomes (up to 4 model results). Excel templates (supplemental materials) were provided for the participants to provide their predictions for the external dataset. The continuous outcome to be predicted was PFS in months, and the dichotomous outcomes were 1-, 2-, and 3-y PFS. Participants were requested to complete a questionnaire to provide the challenge team with some background information on their models. The questionnaire, provided in the supplemental materials, was used to obtain information on feature preprocessing and selection, the machine-learning method used, data balancing (for training), performance evaluations used during training, interval validation (e.g., cross-validation, bootstrapping), and code or tool availability.

Model performance for the external dataset was assessed by the challenge team, who was fully masked to the participants’ identifies, using R, version 4.2.1, with pRoc, version 1.18.5. The main performance criterion to assess regression model performance was the root-mean-square error (RMSE) between predicted and observed PFS, in months. As an additional exploratory FOM, we derived the Pearson correlation coefficient between observed and predicted PFS to understand if there was any association between predicted and observed PFS. For the classification models, the average C-index for 1-, 2-, and 3-y PFS was derived to characterize the model performance.

### Reference Model FOM

For comparison purposes, the SNMMI-AITF challenge team trained and tested 2 simple reference models based on existing knowledge about survival prediction in patients with diffuse large B-cell lymphoma. For both models, only 2 basic features were used as input parameters. For the prediction of PFS, SUV_peak_ and TMTV or their logarithmic-transformed values were used within a generalized linear model. For the prediction of 1-, 2-, and 3-y PFS, the logarithmic-transformed SUV_peak_ and TMTV were fitted using a logistic regression model. After training or fitting these models, a reference FOM was calculated for the external data in the exact way it was done when assessing FOMs for the participants’ models.

## RESULTS

### Datasets

**FIGURE 1.**
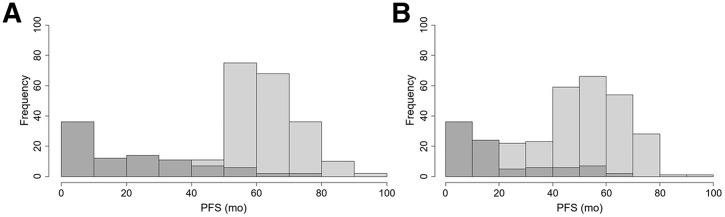
Histogram of PFS by event type for training (A) and testing (B) datasets. Event type 1 was disease progression or death (dark gray); event type 0 (light gray) indicated end of follow-up or study drop out.

Table 1 displays some demographic and clinical information for both training and external datasets. Age, disease stages, health performance status, and lactate dehydrogenase values significantly differed between datasets. Figure 1 indicates the distribution of PFS by event type for both datasets, showing that most PFS events occurred before 3 y, whereas the nonevents occurred later, with a clear higher frequency around 55–60 mo, close to the 5-y end of follow-up for both trials.

**TABLE 1.** Demographic and Disease Stage Information for Training (HOVON-84 Group) and External (PETAL Group) Data

Characteristic	HOVON-84 group (*n* = 294)*	PETAL group (*n* = 339)^†^	*P*
Age (y)	63 ± 13	58 ± 14	<0.001
WHO performance			<0.001
0	169	158	
1	89	148	
2	36	26	
3	0	7	
Ann Arbor stage			<0.001
1	0	65	
2	56	78	
3	88	65	
4	150	131	
LDH value			<0.01
Normal	95	149	
Greater than normal	199	190	
Extranodal involvement			NS
0	176	237	
≥1	118	102	
IPI^‡^			<0.001
1	49	123	
2	71	91	
3	100	83	
4	73	42	

* Demographic information missing for 2 patients.

† Demographic information missing for 1 patient.

‡ International Prognostic Index (IPI) was not available for 1 patient in the training dataset.

WHO = World Health Organization; LDH = lactate dehydrogenase; NS = nonsignificant.

Data represent number of patients, except age, reported as mean ± SD.

### Reference Model FOM

**FIGURE 2.**
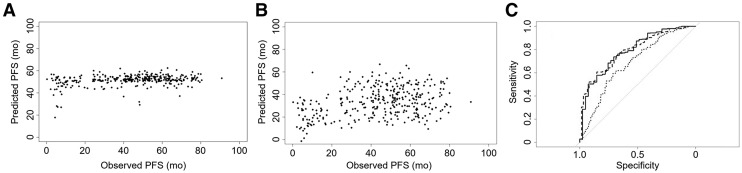
External validation results (all events) using simple reference models for predicting continuous outcomes when model was trained using all event types (A), when model was trained using event type 1 dataset only (B), and when model was trained using dichotomous outcomes for 1-y PFS (solid line), 2-y PFS (dashed line), and 3-y PFS (dotted line) (C).

External validation results obtained using the best-performing reference models are shown in Figure 2. The best-performing linear model to predict continuous PFS outcome based on SUV_peak_ and TMTV showed a strong preference to predict PFS close to the most frequent follow-up time. The RMSE of this model equaled 21.4 mo. As this approach was clearly not meaningful, the simple model was retrained on data for event type 1 (progression of disease or death). The latter approach resulted in a worse RMSE (23.1 mo), but the model showed a somewhat better association between predicted and observed PFS. The optimal logistic regression model to predict dichotomous PFS outcomes using logarithmic-transformed values of both SUV_peak_ and TMTV showed an area under the curve (AUC) of 0.788, 0.789, 0.681 for 1-, 2-, and 3-y PFS (mean AUC, 0.753).

### Summary of Methods Used by Participants

Ninety-one registrations were received before sharing the data. Eventually, external validation results of 19 models for predicting continuous PFS from 15 teams were received. Nine of those teams also provided the SNMMI-AITF challenge team with results from 12 classification models predicting dichotomous 1-, 2-, and 3-y PFS. A questionnaire was sent to registered participants who did not submit results to inquire about their reason for not participating. Of the 30 responses received, 20 indicated a lack of time as the reason for not participating. A variety of other reasons were given by the remaining10 registered participants who did not participate, such as “I lack the expertise to join” or “I already participate in another team.”

There was large variability in the methods applied by the 15 teams for the various steps during continuous outcome model development. An overview of these methods is presented in Table 2 and Supplemental Table 1, showing no clear trends in consistency of methods used among participants, except for internal validation, with 16 of 19 continuous models validated using repeated k-fold cross-validation. Similar observations were seen for the development of the classification models and are summarized in Supplemental Table 2. Of the 31 models submitted (19 continuous and 12 dichotomous), 17 models were made publicly available. To the best of our knowledge, 7 models have been published (supplemental materials), but documentation is quite limited for most models.

**TABLE 2.** Overview of Methods Used During Each Step of Model Development for Best-Performing Models Based on External Validation

Model development step	Continuous model	Classification model
Feature preprocessing/scaling	Logarithmic transformation	Min–max normalization
Feature selection	Lasso and Cox regression	Recursive feature elimination
Dimension reduction	Pairwise elimination	PCA
Machine-learning method	Ensemble boosted tree	Component-wise gradient boosting
Data balancing	None	None
Internal validation	5-fold CV	5-fold CV with nested test
Loss function	RMSE for training loss	C-index

PCA = principal component analysis; CV = cross-validation.

### Results from Masked External Testing

**FIGURE 3.**
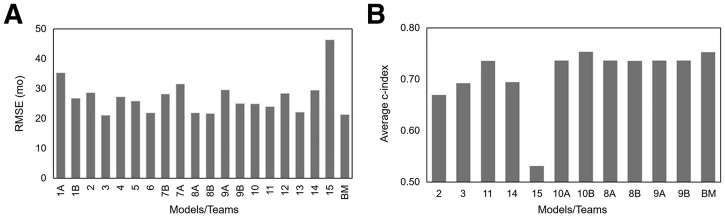
FOMs derived from external dataset for models providing continuous outcome predictions (A) and dichotomous outcome predictions (B). Benchmark (BM) data are reference model results.

**FIGURE 4.**
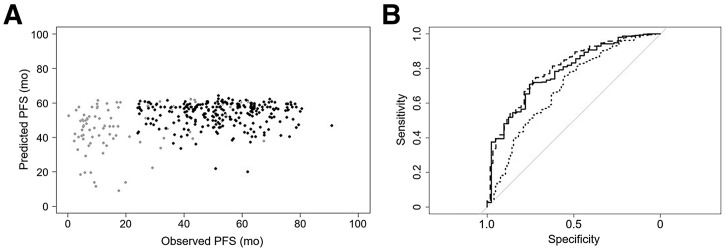
FOMs for best-performing continuous model per event types 1 (gray dots) and 0 (black dots(A). FOMs for best-performing classification model for 1-y PFS (solid line), 2-y PFS (dashed line), and 3-y PFS (dotted line) (B).

For the continuous outcome (Fig. 3A), the observed RMSE ranged from 21.1 to 46.4 mo. Approximately half of the models showed an RMSE value better than that of the reference model performance when the model was trained using event type 1 data (progression of disease or death) only (RMSE, 23.1 mo). Results from the exploratory correlation analysis are shown in Supplemental Figure 1, indicating that the best-performing model based on RMSE was also the model identified as best using correlation coefficients and provided the best association with observed PFS data, making this the winning model (Fig. 4A).

Among all classification models (Fig. 3B), only 1 model showed an average C-index higher than that of the reference classification model. Seven of 12 models showed an average C-index close to 0.75. For one of these models, the results were not provided in the correct format and could not be processed, resulting in 11 evaluable model results. The best-performing model had an average C-index of 0.754, with corresponding receiver operator characteristic curves shown in Figure 4B. Supplemental Table 2 lists the main methods used for each step during development of the best-performing models.

## DISCUSSION

This challenge was created to gain a better understanding of the variability in machine-learning methodology and model performances for predicting outcomes based on a radiomic feature dataset. Our findings suggest that there was large variability in the methods used during model development. Moreover, we found that some of the radiomics-based machine-learning models slightly outperformed the simple reference models, but differences in FOMs were not significant. In fact, several models performed worse in terms of the FOMs than the simple reference models trained by the SNMMI-AITF challenge team.

We observed a bimodal distribution for both the training and external datasets, with event type 1 (progression of disease or death) mainly occurring during the first 3 y, whereas a second peak in the distribution was found around 55 to 60 mo, mainly explained by end of follow-up for most patients. An event occurred for approximately 20% of patients, suggesting a large degree of right censoring in the data. This makes the development of a model that can predict PFS continuously difficult, as most of the reported PFS data do not represent progression of disease. This problem can be avoided by using binary outcomes with classification models and may explain the better results found by classification models. Nine of 15 teams provided results for the classification task, in addition to training a model for continuous outcomes, possibly realizing that the survival data were less suited for continuous outcome predictions.

Both datasets have been used previously to develop a radiomics model using logistic regression (18). Eertink et al. (16,21) developed a radiomics model that included SUV_peak_, TMTV, and a dissemination feature, reflecting the spatial spread of disease (7). The model showed an AUC of the receiver operator characteristic curve of 0.76 for predicting 2-y time to progression using the training dataset. The addition of clinical information to the model improved performance to 0.79. Later, during external testing, Eertink et al. (21) found an AUC of 0.75 for predicting 2-y PFS for the training and testing datasets using a clinical PET model (i.e., a model including both radiomic and clinical information). Recently, Ferrández et al. (18) externally tested a PET-only radiomics model (in addition to a deep-learning model) and reported an AUC of 0.76 for predicting 2-y time to progression for the same dataset as the external dataset in this challenge. The reported C-index values from the 7 best-performing classification models were similar (∼0.74; Fig. 3B).

Several factors may account for the somewhat lower average performance of the challenge models. The challenge FOM was based on the average C-index for 1-, 2-, and 3-y PFS, whereas Eertink et al. (16) and Ferrández et al. (18) used 2-y time to progression and 2-y PFS. The 2-y PFS results for the challenge’s best-performing classification model had a C-index of 0.79, which suggests a slightly better performance for the radiomics model than the 2-y PFS AUC reported by Ferrández et al. (18). As seen in Figure 4, the model performance for 3-y PFS was lower than that seen for 1- and 2-y PFS, which lowered the FOM used in the challenge. Moreover, clinical information was not provided in the challenge’s datasets, which could have further improved performances of the challenge team’s models. Other published models exploiting ^18^F-FDG PET radiomics for PFS prediction in different cohorts of patients with diffuse large B-cell lymphoma never reported performance greater than 0.75 for AUC, time-dependent AUC, or C-index (7,24), suggesting that clinical and ^18^F-FDG PET images may not contain enough information to achieve higher predictive accuracy, regardless of the sophisticated modeling used. The findings of this challenge seem to confirm that radiomics and machine learning are not likely to improve outcome prediction based on baseline ^18^F-FDG PET/CT in patients with diffuse large B-cell lymphoma and that simple uptake metrics, such as SUV, TMTV, and dissemination features, seem to capture most of the prognostic information present in PET images (18). However, for diseases with less-disseminated tumor spread, radiomics and machine learning may have added value and should be evaluated using challenges similar to the one presented here.

Overall, the limited ability of any model to predict PFS may be explained by the definition of the endpoint. Indeed, in our definition, PFS encompassed both lymphoma-related events (progression or relapse) and death unrelated to lymphoma. It is thus suspected that radiomic features pertaining to lymphoma lesions cannot accurately predict deaths unrelated to lymphoma.

### Limitations

A possible limitation of the study datasets is the high number of right-censored data points, posing a challenge in the training and external validation of models using continuous PFS outcomes. This problem can be avoided by using dichotomous outcomes, and this was the reason that participants were allowed to enroll in the challenge for both approaches. However, even with changing continuous outcomes to dichotomous outcomes, right censoring is not fully excluded. For example, the lower AUC seen for 3-y PFS (Fig. 4) was consistently found for all of the submitted models and may be explained by the inclusion of more event-free (nonevent) data within the 3-y PFS interval than using a 2-y PFS interval (Fig. 1), especially for the external validation dataset.

Another limitation of the challenge was the choice of the FOM. Although the RMSE between predicted and observed PFS seems to be a logical choice for continuous outcomes, this FOM does not capture all aspects of model performance. For example, a model that predicts a PFS of 50 mo for all patients would result in a RMSE of 22.0 mo. Clearly, such a model would be useless while a decent RMSE was found, which is not very much worse than the RMSE reported for the best-performing model and within the range of the other observed model performances.

For the classification model, the average C-index (or AUC of the receiver operating curve) for 1-, 2-, and 3-y PFS was used as the FOM. Although this is a frequently used metric to report model performance for binary outcomes, data imbalance may also result in optimistic results when predictions tend to be biased toward the majority class.

Moreover, we did not include clinical or demographic information in the training and testing datasets. Including this information during model development may have improved model performance. In addition, we did not compare the results with the clinically used International Prognostic Index. Previous studies have shown that prognostic models based solely on PET uptake metrics performed similarly to models including clinical information and outperformed the International Prognostic Index for several external datasets (18).

In addition, both datasets were multicentric. These data were used in previous studies, and careful data curation and quality control were performed during image collection and processing to ensure sufficient quality of the images and derived data (18,21). However, we cannot rule out some heterogeneity in image quality among the patients’ scans. Yet, the datasets have been used previously for external validation of radiomics and deep-learning methods, and in all cases, the PETAL testing dataset consistently provided comparable results to those seen during cross-validation training of the HOVON-84 training dataset (18,21).

Finally, the radiomic features were generated using a specific analysis pipeline and a specific set of feature-extraction settings based on earlier publications. Yet, it could well be that several radiomic features would have benefitted from the use of different settings, such as a different intensity discretization or different segmentation methods. In any radiomics study, there are many settings that can be chosen or optimized, making the development and use of radiomic analysis for clinical applications particularly challenging. In this challenge, we opted to apply more commonly used settings, similar to those used previously (16).

## CONCLUSION

The SNMMI-AITF radiomics challenge 2024 aimed to compare machine-learning models for predicting survival in patients with diffuse large B-cell lymphoma using a baseline ^18^F-FDG PET/CT radiomics dataset. We observed that, although participants used a large variety of methods during each step of machine-learning model development, radiomics-based machine-learning models provided relatively similar performances to those of simple models using commonly used metrics, such as SUV_peak_ and TMTV. The best-performing classification model showed only a slightly better (nonsignificant) performance for 2-y PFS (C-index) for the external validation dataset than that of a previously reported model that included SUV_peak_, TMTV, and a dissemination feature. This suggests that additional nonradiomic information may be needed to achieve higher predictive accuracy. This challenge was thus more related to radiomics data and whether it was sufficient to predict the outcome of interest, rather than the modeling methods used to analyze this information.

## DISCLOSURE

Josée Zijlstra received financial support for clinical trials from Roche, Gilead, and Takeda. Pieternella Lugtenburg received financial support for clinical trials from Takeda and Roche. Ronald Boellaard has a research grant from Siemens Healthineers. No other potential conflict of interest relevant to this article was reported.

## Notes

KEY POINTS **QUESTION:** The choice and optimization of radiomics-based machine learning models are not straightforward processes, and would this have a large impact on model performance as suggested by the large variation in observed FOMs among several participating teams? **PERTINENT FINDINGS:** Radiomics-based machine-learning models do not necessarily outperform simple linear or logistic regression models using standard uptake metrics, such as SUV and TMTV. **IMPLICATIONS FOR PATIENT CARE:** Benchmark models using only SUV and TMTV (or their logarithmic-transformed values) are useful, and their performance should be reported, along with results from any radiomics-based machine-learning model, to determine if radiomics-based models provide added value.
